# The relationship between disease activity, sleep, psychiatric distress and pain sensitivity in rheumatoid arthritis: a cross-sectional study

**DOI:** 10.1186/ar2842

**Published:** 2009-10-29

**Authors:** Yvonne C Lee, Lori B Chibnik, Bing Lu, Ajay D Wasan, Robert R Edwards, Anne H Fossel, Simon M Helfgott, Daniel H Solomon, Daniel J Clauw, Elizabeth W Karlson

**Affiliations:** 1 Division of Rheumatology, Immunology and Allergy, Brigham and Women's Hospital, 75 Francis Street, PBB-B3, Boston, MA, 02115, USA; 2 Pain Management Center, Brigham and Women's Hospital, 850 Boylston Street, Chestnut Hill, MA, 02467, USA; 3 Chronic Pain and Fatigue Center, University of Michigan Medical School, Domino's Farms, Lobby M, PO Box 385, 24 Frank Lloyd Wright Drive, Ann Arbor, MI, 48106, USA

## Abstract

**Introduction:**

Despite recent advances in anti-inflammatory therapy, rheumatoid arthritis (RA) patients continue to rate pain as a priority. The etiology of RA pain is likely multifactorial, including both inflammatory and non-inflammatory components. In this study, we examine the association between disease activity, sleep, psychiatric distress and pain sensitivity in RA.

**Methods:**

Fifty-nine female RA patients completed questionnaires and underwent pressure pain threshold testing to assess hyperalgesia/allodynia at joint and non-joint sites. Blood samples were taken to measure C-reactive protein (CRP). The association between disease activity, sleep problems, psychiatric distress and pain threshold was assessed using Pearson/Spearman correlations and multivariable linear regression. Disease activity levels, sleep problems and psychiatric distress were compared between RA patients with fibromyalgia and RA patients without fibromyalgia.

**Results:**

In unadjusted analyses, CRP was not correlated with pain threshold, but tender joint count was inversely correlated with pain threshold at all sites (*P *≤ 0.004). Sleep problems were associated with low pain threshold at all sites (*P *≤ 0.0008). Psychiatric distress was associated with low pain threshold at the wrist and thumbnail (*P *≤ 0.006). In multivariable linear regression models, CRP was inversely associated with wrist pain threshold (*P *= 0.003). Sleep problems were inversely associated with pain threshold at all sites (*P *≤ 0.01), but psychiatric distress was not. Despite differences in pain threshold, CRP levels and sleep problems between RA patients with fibromyalgia and those without fibromyalgia, associations between these variables did not change when patients with fibromyalgia were excluded.

**Conclusions:**

Multivariable models are essential in analyses of pain. Among RA patients, inflammation is associated with heightened pain sensitivity at joints. In contrast, poor sleep is associated with diffuse pain sensitivity, as noted in central pain conditions such as fibromyalgia. Future studies examining pain sensitivity at joint and non-joint sites may identify patients with different underlying pain mechanisms and suggest alternative approaches to treating RA pain.

## Introduction

Rheumatoid arthritis (RA) is a chronic inflammatory disease that causes significant pain. Despite the development of effective medications to treat inflammation, pain remains a priority for RA patients [1,2]. Many RA patients report pain at non-joint sites, suffering from ongoing pain even when inflammation appears to be well controlled [3].

To date, most studies of pain in RA have focused on clinical pain severity. Kojima and colleagues recently reported independent effects of depression severity and inflammation on perceived pain in RA patients, emphasizing the complex relation between pain, inflammation and psychiatric distress [4]. An earlier, cross-sectional study showed strong associations between clinical pain severity, sleep disturbance and mood [5]. Although these studies provided critical information regarding clinical factors associated with pain severity, they did not quantify the patients' underlying pain sensitivity or yield information about pain mechanisms.

Pain sensitivity is measured by responses to experimental stimuli such as pressure. High pain thresholds represent low pain sensitivity, whereas low pain thresholds represent high pain sensitivity [6-8]. Increased pain in response to normally painful stimuli is termed hyperalgesia, whereas pain in response to normally non-painful stimuli is termed allodynia. Pain thresholds may be measured at different locations to yield a comprehensive assessment of pain sensitivity at joint and non-joint sites [9].

Although it is well-documented that RA patients have lower pain thresholds than healthy controls [6,10-16], little is known about the factors associated with low pain thresholds and whether these factors impact pain thresholds on a peripheral, local level (i.e. at the joints) or on a central, widespread level. No studies of RA patients have incorporated assessments of pain threshold with comprehensive assessments of sleep problems and psychiatric distress, although these factors are: associated with reported pain severity [17-22] and low pain threshold [23-27] among healthy individuals and individuals with non-inflammatory pain syndromes; prevalent among the RA population [5,28-31]; and associated with reported pain severity among RA patients [32-37].

As differences in pain sensitivity may shape the course of pain complaints and influence treatment decisions [38], it is important to understand the factors associated with enhanced pain sensitivity. In this study, we examined the relation between disease activity, sleep, psychiatric distress and pain threshold in RA patients. We hypothesized that both inflammatory and non-inflammatory factors are important mediators of pain sensitivity. Specifically, we hypothesized that objective measures of disease activity, such as C-reactive protein (CRP), are associated with pain threshold at RA-affected joints but not at sites distant from joints. We hypothesized that sleep and psychiatric distress are associated with decreased pain threshold at all sites, as is seen in chronic, non-inflammatory pain conditions such as fibromyalgia.

## Materials and methods

### Patients

Female RA patients were recruited from an academic medical institution from February to July 2008. Inclusion criteria were: RA diagnosed by a board-certified rheumatologist and female sex. The study was limited to women because men and women have different pain thresholds and must be analyzed separately. Because 82% of RA patients at this academic institution are women, we were underpowered to study men. Participants were excluded if they had taken opiate medications within one week of the study. The study was approved by the Partners Institutional Review Board. All participants provided written informed consent.

### Pressure pain threshold measurements

Participants wore large gloves to blind the investigator to disease activity in the hands. A rheumatologist (YCL) trained in algometry assessed pressure pain threshold using a Wagner FPK 20 algometer (Wagner Instruments, Greenwich, CT, USA) at joints (wrists), sites close to joints (thumbnails) and sites distant from joints (trapezius muscles). The investigator explained the procedure using a standard script. A trial run was performed to accustom participants to the procedure. Testing began at the thumbnails, continued at the wrists and ended at the trapezius muscles. All measurements were performed bilaterally, right side first. The investigator increased the pressure at a rate of 1 kg/s to a maximum of 11 kg. The pain threshold was defined as the pressure at which participants first indicated pain. After a five minute rest/equilibrium period, testing was repeated.

### Assessment of clinical variables

Disease activity was measured by high sensitivity CRP, swollen and tender joint counts and the disease activity score in 28 joints (DAS28). High sensitivity CRP was chosen because the range of CRP concentrations varies greatly among RA patients, and this assay is capable of measuring both low and high concentrations of CRP. Sleep problems were quantified using the Sleep Problems Index II of the Medical Outcomes Study (MOS) sleep scale, a validated, 12-item questionnaire that assesses sleep problems in chronically ill populations [39]. Psychiatric distress was measured using the Hospital Anxiety and Depression Scale (HADS), a validated 14-item questionnaire that assesses depression and anxiety in physically ill patients [40]. Participants also completed the Brief Pain Inventory - short form (BPI-sf), a validated, nine-question survey, to characterize clinical pain severity [41].

Disease duration and medication use were obtained by patient self-report. Rheumatoid factor (RF) and anti-cyclic citrullinated peptide (anti-CCP) levels were obtained from chart review. Participants were classified as RF positive if they had a RF level of 15 IU/ml or higher (nephelometry) or if their rheumatologist documented RF seropositivity in the medical chart. Participants were classified as anti-CCP positive if they had an anti-CCP level of 5 U/ml or higher (second-generation Axis Shield DIASTAT™ ELISA assay) or if their rheumatologist documented anti-CCP seropositivity in the medical chart. All data were double-entered by two individuals and checked against each other for accuracy.

### Statistical testing

The reproducibility of algometry measurements was assessed by intraclass correlations (ICCs) and percent change in pain threshold. For all analyses of association, we used the mean pain threshold between the first and second trials and the left and right side. The distributions of all variables were examined. Means and standard deviations (SDs) were calculated for normally distributed variables. Minimum, median and maximum values were reported for variables that were not normally distributed.

Plots of pain threshold versus clinical variables were examined for non-linear relations. Pearson and Spearman coefficients were used to examine the unadjusted associations between clinical variables and pain threshold at each site. Correlation coefficients between -0.3 and 0.3 were interpreted as no to low association. Coefficients between -0.3 and -0.6 were considered moderate inverse associations, and coefficients below -0.6 suggested strong inverse associations.

In multivariable linear regression models, we examined the combined effect of disease activity, sleep problems and psychiatric distress on pain threshold at each site. Because composite measures (i.e., DAS28-CRP) are influenced by subjective measures of tenderness unrelated to inflammation [38], we chose CRP as the measure of disease activity in these models. We chose the total HADS score to represent psychiatric distress because it assesses both depression and anxiety. We adjusted all analyses for age, given previous studies suggesting an association between pain threshold and age [42]. We also included covariates associated with pain threshold at *P *< 0.1 in unadjusted analyses. Non-linear relations were assessed by adding higher order polynomials to the final multivariable model. Given the strong association between total HADS score and MOS sleep score, *post-hoc *analyses were performed to determine whether the results would change if either variable were removed from the model.

Because psychiatric distress and sleep problems may identify a subset of RA patients with fibromyalgia rather than pertain to RA patients as a whole, we calculated the median total HADS score and median MOS sleep score among RA patients with fibromyalgia (defined by ≥ 11 tender points) and compared them in tabular form to the group of RA patients with less than 11 tender points. We also compared the mean pain thresholds of RA patients with and without 11 or more tender points. Formal statistical testing was not performed due to the small number of patients with 11 or more tender points. To further assess these relations, we inserted two-way interaction terms in the final models to determine whether the relations between inflammation, sleep problems, psychiatric distress and pain threshold differed based on the presence of 11 or more tender points. To confirm that these relations remained the same among RA patients who had less than 11 tender points, we ran the final model, excluding participants with 11 or more tender points.

The strength of association was assessed using regression coefficients (β) and *P *values. The β coefficient represents the change in outcome given a one unit change in the predictor, holding all other variables constant. The threshold for significance was set as a two-tailed *P *< 0.05. All analyses were performed using the SAS 9.1 software package (SAS Institute, Cary, NC, USA).

## Results

### Patient characteristics

Fifty-nine female RA patients participated in this study (Table 1). Mean age was 61.0 ± 15.2 years and 89.8% were Caucasian. The median BPI-sf pain severity score was 3.0, and 37.3% had wrist swelling consistent with synovitis. Of the 59 patients, 67.8% were treated with a synthetic disease-modifying anti-rheumatic drug (DMARD), 62.7% were treated with a biologic DMARD, and 40.7% were treated with both synthetic and biologic DMARDs.

**Table 1:** Characteristics of 59 rheumatoid arthritis patients

Characteristics	Value
Mean age in years (SD)	61.0 (15.2)
Median disease duration in years (min, max)	9.5 (1, 58.5)
Caucasian (N, %)	53 (89.8)
Rheumatoid factor positive (N, %)	32 (68.1)^1^
Anti-cyclic citrullinated peptide positive (N, %)	26 (86.7)^1^
Median tender joint count (min, max)	4.0 (0, 26)
Median swollen joint count (min, max)	4.0 (0, 24)
Median C-reactive protein in mg/L (min, max)	2.6 (0, 17.9)
Mean DAS28-CRP (SD)	3.4 (1.3)
Median Brief Pain Inventory - short form pain severity score (min, max)	3.0 (0, 10)
Median Medical Outcomes Study sleep score (min, max)	33.1 (0, 88.9)
Median Hospital Anxiety and Depression Scale anxiety score (min, max)	4.0 (0, 17)
Median Hospital Anxiety and Depression Scale depression score (min, max)	3.0 (0, 13)
Median Hospital Anxiety and Depression Scale total score (min, max)	7.0 (0, 29)
Synthetic disease modifying anti-rheumatic drug use (N, %)	40 (67.8)
Biologic disease modifying anti-rheumatic drug use (N, %)	37 (62.7)
Combination therapy with both a synthetic and a biologic disease modifying anti-rheumatic drug (N, %)	24 (40.7)
Non-steroidal anti-inflammatory drug use (N, %)	23 (39.0)
Corticosteroid use (N, %)	18 (30.5)

^1 ^Percent of patients with recorded measurements (anti-cyclic citrullinated peptide: n = 30; rheumatoid factor: n = 47)

CRP = C-reactive protein; DAS28 = Disease Activity Score in 28 joints; SD = standard deviation.

### Pressure pain thresholds

Mean pain thresholds were lowest at the trapezius muscles (right: 4.3 ± 1.9 kg/cm^2^; left: 4.4 ± 2.2 kg/cm^2^) and highest at the thumbnails (right: 5.5 ± 2.7 kg/cm^2^; left: 5.3 ± 2.6 kg/cm^2^). Percent change in pain threshold between tests 1 and 2 ranged from -8.3 ± 29.7% to 3.6 ± 28.2%. ICCs ranged between 0.80 and 0.92 (Table 2). Correlations between pain thresholds and reported pain severity, measured by the BPI-sf pain severity score, ranged from -0.25 at the trapezius to -0.35 at the wrists.

**Table 2:** Reproducibility of pressure pain threshold measurements

Site	Pressure pain threshold		
	
	Mean ± SD (kg/cm^2^)	% change^1^ ± SD	Intraclass correlations
Right wrist	5.0 ± 2.1	-8.3 ± 29.7	0.80
Left wrist	5.0 ± 2.1	-4.0 ± 28.7	0.85
Right thumbnail	5.5 ± 2.7	3.6 ± 28.2	0.87
Left thumbnail	5.3 ± 2.6	-0.3 ± 25.0	0.90
Right trapezius	4.3 ± 1.9	-1.3 ± 27.9	0.83
Left trapezius	4.4 ± 2.2	-0.7 ± 22.2	0.92

^1 ^Percentage change = (pain threshold_trial 1 _- pain threshold_trial 2_)/pain threshold_trial 2 _× 100%

SD = standard deviation.

### Unadjusted associations between clinical variables and pressure pain thresholds

#### Disease activity variables

CRP was not significantly associated with pain threshold at any site. Swollen joint count was inversely associated with wrist pain threshold (r = -0.37, *P *= 0.004) but not with pain threshold at the thumbnails or trapezius muscles. Tender joint count was inversely associated with pain threshold at all sites (wrist: r = -0.49, r = 0.0001; thumbnail: r = -0.37, *P *= 0.004; trapezius: r = -0.36, *P *= 0.006), and DAS28-CRP was associated with wrist and thumbnail pain threshold (wrist: r = -0.42, *P *= 0.002; thumbnail: r = -0.29, *P *= 0.03; Table 3).

**Table 3:** Univariate association between clinical variables and pain threshold at the wrists, thumbnails and trapezius muscles

Variable	Wrist pressure pain threshold		Thumbnail pressure pain threshold		Trapezius pressure pain threshold	
			
	r	*P *value	r	*P *value	r	*P *value
Disease activity variables						
C-reactive protein	-0.24	0.09	-0.12	0.39	0.07	0.61
Swollen joint count	-0.37	0.004	-0.18	0.16	-0.14	0.30
Tender joint count	-0.49	0.0001	-0.37	0.004	-0.36	0.006
DAS28-CRP	-0.42	0.002	-0.29	0.03	-0.20	0.16

Sleep						
Medical Outcomes Study Sleep	-0.51	< 0.0001	-0.44	0.0005	-0.43	0.0008

Psychosocial Variables						
HADS - Total	-0.42	0.0009	-0.35	0.006	-0.22	0.09
HADS - Depression	-0.37	0.004	-0.33	0.01	-0.19	0.15
HADS - Anxiety	-0.34	0.008	-0.27	0.04	-0.20	0.13

CRP = C-reactive protein; DAS28 = Disease Activity Score in 28 joints; HADS = Hospital Anxiety and Depression Scale.

#### Sleep/psychiatric distress

The MOS sleep problems index II was inversely associated with pain threshold at all sites (wrists: r = -0.51, *P *< 0.0001; thumbnails: r = -0.44, *P *= 0.0005; trapezius: r = -0.43, *P *= 0.0008). The total HADS score, HADS depression score and HADS anxiety score were significantly associated with pain threshold at the wrists (total: r = -0.42, *P *= 0.0009; depression: r = -0.37, *P *= 0.004; anxiety: r = -0.34, *P *= 0.008) and thumbnails (total: r = -0.35, *P *= 0.006; depression: r = -0.33, *P *= 0.01; anxiety: r = -0.27, *P *= 0.04) but not at the trapezius muscles (Table 3).

#### Other variables

Participants taking biologic DMARDs had significantly lower mean wrist pain thresholds than participants not taking biologic DMARDS (4.5 ± 1.9 kg/cm^2 ^vs. 5.8 ± 1.9 kg/cm^2^; *P *= 0.01). Non-steroidal anti-inflammatory drug (NSAID) use, synthetic DMARD use or corticosteroid use was not associated with pain threshold at any site. There was no association between RF status, anti-CCP status or disease duration and pain threshold at any site.

### Multivariable associations between clinical variables and pressure pain threshold

CRP was significantly inversely associated with wrist pain threshold in multivariable analyses adjusted for age, sleep problems, psychiatric distress and biologic DMARD use (β = -0.15, *P *= 0.003). The association between CRP and thumbnail pain threshold was borderline significant (β = -0.13, *P *= 0.05). CRP was not significantly associated with trapezius pain threshold. In multivariable models, the MOS sleep problems index II was significantly inversely associated with pain threshold at all sites (wrists: β = -0.04, *P *= 0.007; thumbnails: β = -0.06, *P *= 0.002, trapezius: β = -0.04, *P *= 0.01), but the total HADS score was not significantly associated with pain threshold at any site (Table 4).

**Table 4:** Independent association between clinical variables and pain threshold (multivariable linear regression analyses)

Variables	Wrist pain threshold^1^		Thumbnail pain threshold^1^		Trapezius pain threshold^1^	
			
	β^2^	*P *value	β^2^	*P *value	β^2^	*P *value
C-reactive protein	-0.15	0.003	-0.13	0.05	-0.03	0.55
Medical Outcomes Study Sleep Problems Index II	-0.04	0.007	-0.06	0.002	-0.04	0.01
Hospital Anxiety and Depression Scale Total Score	-0.03	0.48	0.01	0.80	-0.009	0.85

^1 ^Separate models were run for wrist pain threshold, thumbnail pain threshold and trapezius pain threshold. All models included the three variables above (C-reactive protein, Medical Outcomes Study Sleep Score, Hospital Anxiety and Depression Scale Total Score) and were adjusted for age and biologic disease-modifying anti-rheumatic drug use.

^2 ^The β-coefficient represents the change in the outcome (i.e., pain threshold) given a one unit change in the predictor, holding all other variables constant. β-coefficients of different predictors cannot be directly compared because a one unit change in one predictor (i.e., 1 mg/ml change in CRP) may be quite different from a one unit change in another predictor (i.e., one unit on the Medical Outcomes Study Sleep Score.

When the total HADS score was removed from the multivariable model, the β-estimates for CRP and the MOS sleep problems index II did not change significantly. When the MOS sleep problems index II was removed from the multivariable model, the total HADS score became significantly associated with pain threshold at all sites (wrist: β = -0.11, *P *= 0.001; thumbnails: β = -0.09, *P *= 0.04; trapezius: β = -0.08, *P *= 0.04). CRP remained significantly associated with wrist pain threshold (β = -0.13, *P *= 0.01).

### Subgroup analyses of RA patients with and without 11 or more tender points

**Figure 1:**
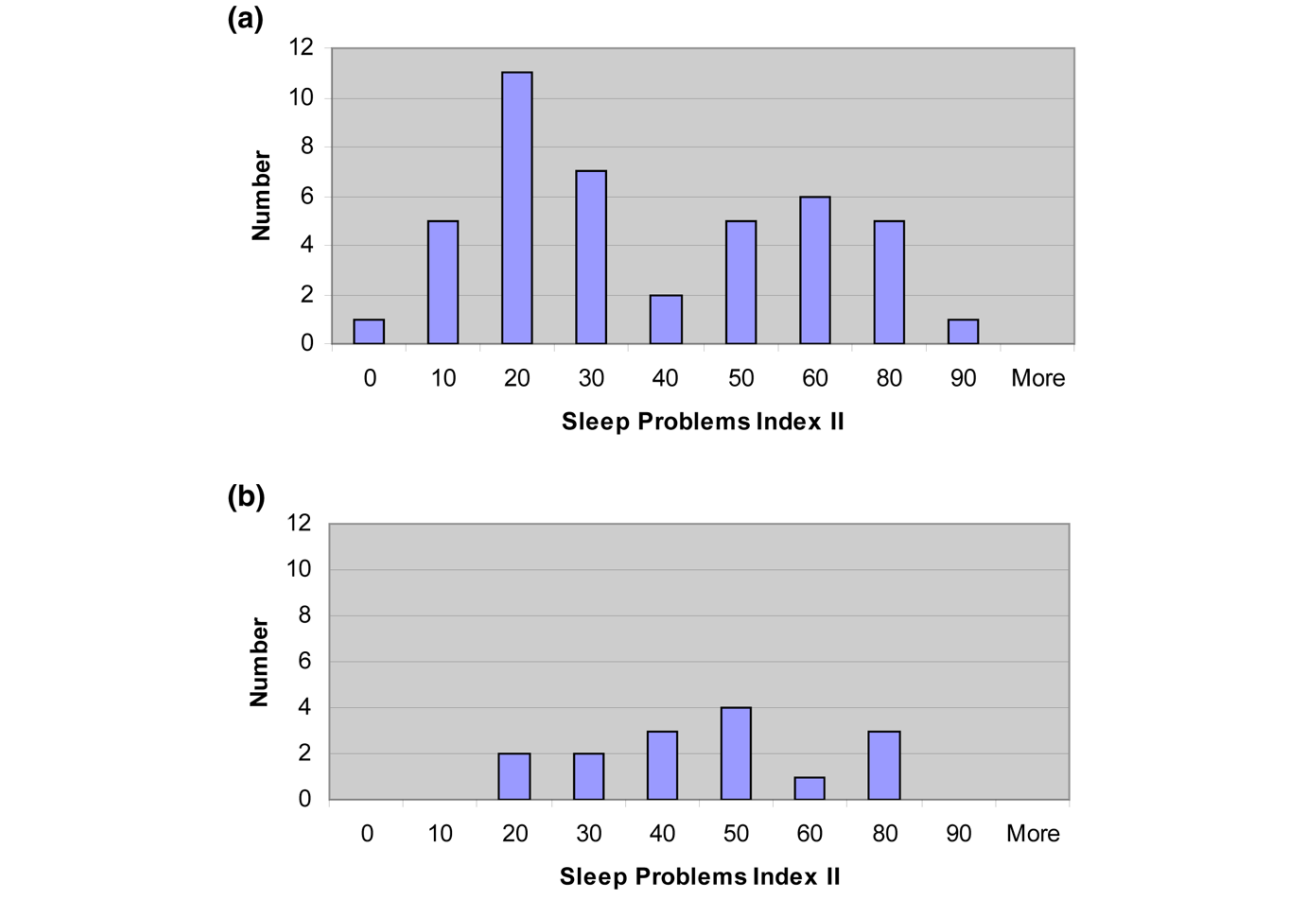
Histograms of the Medical Outcomes Study Sleep Problems Index II among RA patients. **(a) **Patients with less than 11 tender points (n = 43). **(b) **Patients with 11 tender points or more (n = 15).

**Figure 2:**
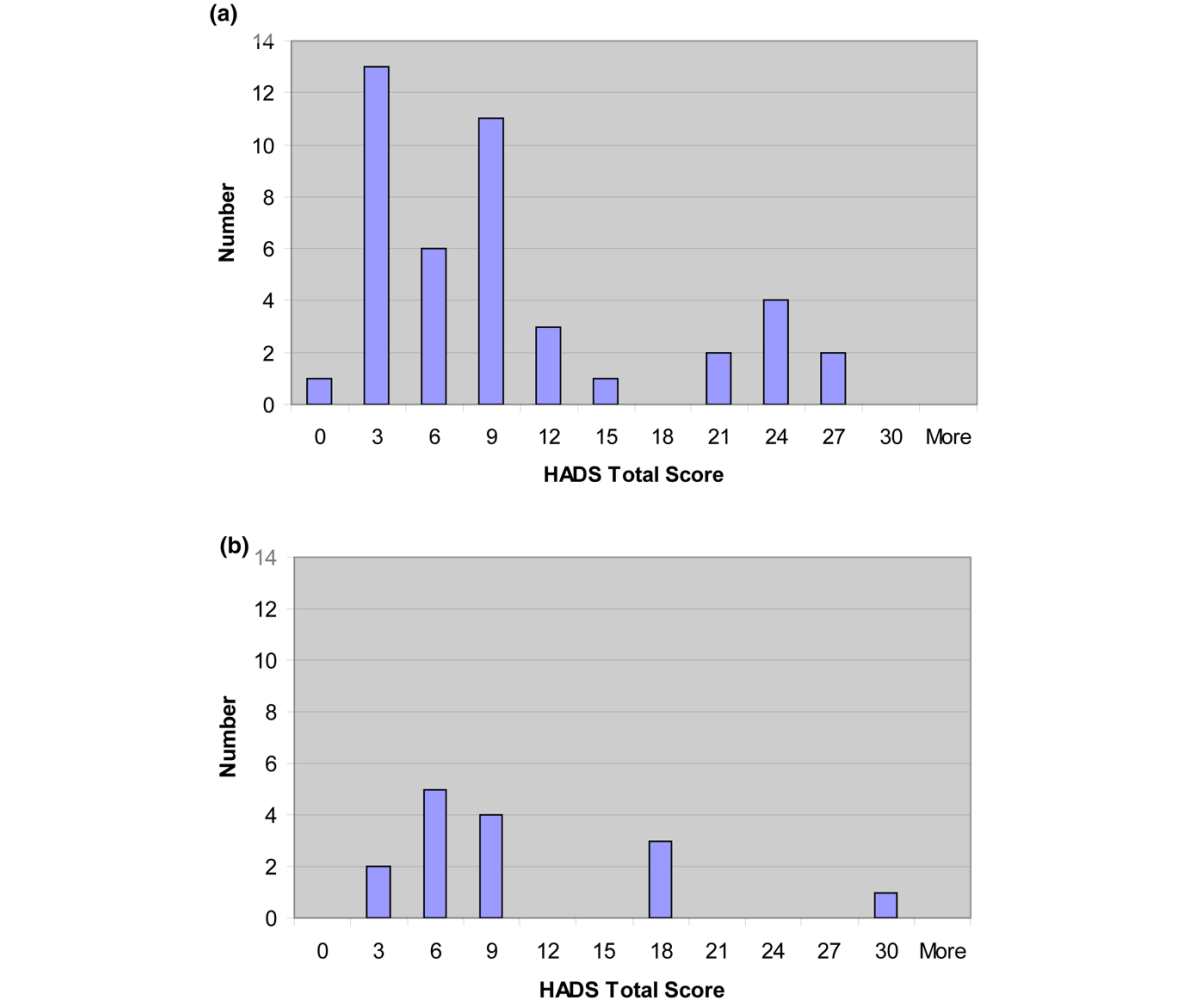
Histogram of the Hospital Anxiety and Depression Scale (HADS) Total Score among RA patients. **(a) **Patients with less than 11 tender points (n = 43). **(b) **Patients with 11 tender points or more (n = 15).

RA patients with 11 or more tender points had more tender joints, greater BPI-sf average pain severity scores and more sleep problems than RA patients with less than 11 tender points (Table 5 and Figure 1). CRP levels also appeared higher among RA patients with 11 or more tender points. Swollen joint counts and measures of psychiatric distress were similar among the two groups (Table 5 and Figure 2).

**Table 5:** Clinical characteristics of RA patients with and without fibromyalgia (defined by ≥ 11 tender points)

Characteristics	RA (n = 43)	RA + FM (n = 15)
Median tender joint count (min, max)	3.0 (0, 26)	7.0 (0, 21)
Median swollen joint count (min, max)	4.0 (0, 24)	4.0 (0, 23)
Median C-reactive protein in mg/L (min, max)	2.4 (0, 17.9)	3.7 (0, 10.6)
Mean DAS28-CRP (SD)	3.2 (1.3)	3.9 (1.2)
Median Brief Pain Inventory - short form pain severity score (min, max)	2.0 (0, 9)	5.0 (2, 10)
Median Medical Outcomes Study sleep score (min, max)	22.8 (0, 88.9)	40.6 (13.3, 68.9)
Median Hospital Anxiety and Depression Scale anxiety score (min, max)	4 (0, 15)	3 (0, 17)
Median Hospital Anxiety and Depression Scale depression score (min, max)	3 (0, 13)	2 (0, 12)
Median Hospital Anxiety and Depression Scale total score (min, max)	7 (0, 27)	7 (1, 29)

CRP = C-reactive protein; DAS28-CRP = Disease Activity Score in 28 joints; FM = fibromyalgia; RA = rheumatoid arthritis; SD = standard deviation.

Compared with RA patients with less than 11 tender points, RA patients with 11 or more tender points had lower pain thresholds at all sites (Table 6). There was no evidence for two-way interactions between the presence of 11 or more tender points and either CRP, the MOS sleep problems index II or the HADS total score. When RA patients with 11 or more tender points were excluded from the analysis, CRP remained significantly associated with pain threshold at the wrists (β = -0.15, *P *= 0.003). Sleep problems remained significantly associated with pain threshold at all sites (wrists: β = -0.05, *P *= 0.0009; thumbnails: β = -0.05, *P *= 0.009, trapezius: β = -0.04, *P *< 0.05).

**Table 6:** Pain thresholds of RA patients with and without fibromyalgia (defined by ≥ 11 tender points)

Characteristics	RA (n = 43)	RA + FM (n = 15)
Mean wrist pain threshold (SD)	5.2 (2.0)	4.4 (1.8)
Mean thumbnail pain threshold (SD)	5.9 (2.5)	4.3 (2.3)
Mean trapezius pain threshold (SD)	4.6 (2.1)	3.6 (1.6)

FM = fibromyalgia; RA = rheumatoid arthritis; SD = standard deviation.

## Discussion

To our knowledge, this is the first study to show an inverse association between CRP and joint pain threshold in RA. Our study differed from previous studies because it focused on pain threshold rather than clinical pain severity, and included assessments of potential non-inflammatory predictors of pain threshold, such as sleep problems and psychiatric distress. Although unadjusted analyses did not reveal significant associations between CRP and pain threshold, multivariable analyses revealed a strong inverse association between CRP and wrist pain threshold. These results suggest that variables, such as sleep problems and psychiatric distress, may confound the association between wrist pain threshold and CRP, emphasizing the need for multivariable models when studying complex outcomes such as pain.

The pattern of association between clinical variables and pain threshold at joint and non-joint sites provides novel insights regarding pain mechanisms. Peripheral mechanisms, such as peripheral sensitization, are characterized by local areas of hyperalgesia/allodynia in response to experimental induction of inflammation [43,44], whereas central mechanisms have widespread effects, involving both joint and non-joint sites. Peripheral sensitization has been demonstrated in animals, healthy individuals and individuals with RA [45], but the role of central pain processing in RA has been largely understudied.

Studies involving healthy individuals and individuals with non-inflammatory pain syndromes (i.e., fibromyalgia) have identified two main mechanisms of widespread pain sensitivity: central sensitization and loss of diffuse noxious inhibitory control [46,47]. Central sensitization includes an acute phase involving sensitization of nociceptors in the spinal cord and a late phase involving diffuse transcriptional changes in the central nervous system [46,47]. Loss of diffuse noxious inhibitory controls is characterized by diffuse hyperalgesia/allodynia due to impairment of the descending pathways that normally induce analgesia [47]. Evidence for central pain processing mechanisms in RA include a small study (n = 12) showing altered regional cerebral blood flow in the prefrontal cortex, cingulofrontal transition cortex and anterior cingulated cortex among RA patients exposed to painful heat stimuli [48].

In our study, CRP was not associated with trapezius pain threshold, arguing against an effect of systemic CRP on widespread pain threshold. However, CRP was strongly associated with wrist pain threshold. This observation is consistent with peripheral sensitization, leading to hyperalgesia at inflamed joints. The strength of association between CRP and pain threshold decreased as the distance between RA-affected joints and the site of pain threshold testing increased. This gradient effect may be due to either the acute phase of central sensitization, characterized by hyperalgesia extending outside the area of direct inflammation [46], or a gradient in local inflammatory mediators. We did not directly measure local inflammatory mediators, but others have shown that serum CRP levels reflect synovial fluid concentrations of inflammatory cytokines [49,50].

These results are contrary to previous studies reporting no association between inflammatory markers and pain threshold [6,12,51] or a direct association between inflammatory markers and pain threshold [52]. These inconsistencies may be due to differences in the sites of testing (i.e. joints vs. non-joint sites) and/or confounding by other variables, such as sleep problems and psychiatric distress.

Although CRP was only significantly associated with pain threshold near RA-affected joints, sleep problems were associated with low pain thresholds at all sites. These findings are consistent with a previous study showing an association between sleep disturbance and clinical pain severity among RA patients [5]. The widespread pattern of association suggests a central mechanism linking sleep disturbance and hyperalgesia/allodynia. This hypothesis is supported by recent studies showing that sleep deprivation (forced awakenings) and short sleep duration are associated with impaired diffuse noxious inhibitory controls among healthy women [22] and individuals with temporomandibular joint disorder [53]. These findings are particularly important given the high prevalence of sleep disturbance among RA patients [5].

In contrast to a previous population-based study [23], our study did not reveal a significant association between psychiatric distress and pain thresholds, after adjustment for sleep problems. However, psychiatric distress was significantly associated with pain threshold at the wrists and thumbnails in unadjusted analyses, and psychiatric distress was significantly associated with pain threshold at all sites in multivariable models that did not adjust for sleep problems. These results likely reflect the strong correlation between sleep and psychiatric distress (r = 0.65). Although pain thresholds were more strongly associated with sleep problems than psychiatric distress in this study, it is not possible to untangle the cause-effect relations due to the cross-sectional design.

The co-occurrence of heightened pain sensitivity, sleep problems and psychiatric distress is common among patients with fibromyalgia, a chronic widespread pain condition that affects approximately 17.1% of RA patients [54]. Given the high prevalence of fibromyalgia among RA patients and the known associations between low pain thresholds, sleep problems and psychiatric distress among fibromyalgia patients, it is possible that our results were driven by the subgroup of patients with fibromyalgia. Consistent with previous studies, our study indicated that RA patients with 11 or more tender points had increased sleep problems and lower pain thresholds than RA patients with less than 11 tender points. However, the associations between pain threshold, CRP, sleep problems and psychiatric distress remained the same after excluding patients with 11 or more tender points from the analysis.

Similarly, we did not find any evidence for effect modification by fibromyalgia on the association between pain threshold and either CRP, the MOS sleep problems index II or the HADS total score. These results were not surprising given recent studies suggesting that widespread pain is a continuous spectrum rather than a discrete entity [55,56]. Scores from the Symptom Intensity Scale, a validated instrument designed to quantify widespread pain, are linearly associated with sleep disturbance, depression, muscle pain and a variety of demographic and sociodemographic factors [55]. Although sleep disturbance is associated with widespread pain, there is no evidence that sleep disturbance has a differential effect on pain threshold among patients with fibromyalgia compared with those without fibromyalgia. However, we cannot exclude the possibility that we were underpowered to see an effect.

This study is limited by its cross-sectional design, which makes it impossible to determine the directionality of associations. While it is accepted that inflammation can lead to hyperalgesia/allodynia in animal models and healthy humans [43,44], this relation may be reversed, or even bidirectional, in RA patients. For example, a recent study showed that TNF-α levels increased after RA patients were exposed to painful experimental stimuli. This phenomenon did not occur in healthy controls [10].

The direction of association between pain threshold and sleep problems is also unclear. Among fibromyalgia patients, path analyses suggest that sleep predicts pain and not vice versa [57]. However, RA pain differs from fibromyalgia pain because RA pain frequently has an inflammatory component. Localized, inflammatory pain may cause sleep disturbance, which, in turn, may lead to greater diffuse pain sensitivity, but without longitudinal data, we were unable to test this hypothesis.

Other limitations include: potential confounding by medications; small sample size; and the lack of a control group. Although we excluded individuals taking opiates, participants could continue to take other medications that may affect pain thresholds. Use of synthetic DMARDs, corticosteroids and NSAIDs were not associated with pain threshold in univariate analyses, but sample sizes were small, limiting the power to detect an association. Without a control group, we could not discern whether the association between CRP and pain threshold at/near joint sites is specific to RA or whether this association also occurs in healthy individuals or individuals with inflammatory diseases that do not preferentially affect joints. Additional studies are necessary to examine the associations between disease activity, sleep, psychiatric distress and pain sensitivity in a larger cohort of RA patients over time. Mechanistic studies involving advanced quantitative sensory tests may also provide insight regarding the cause of increased pain sensitivity in RA patients.

## Conclusions

In this study, CRP was inversely associated with pain threshold at the wrists, consistent with peripheral sensitization. Sleep problems were inversely associated with pain threshold at all sites, suggesting a defect in central pain processing. The association between CRP and pain threshold was only evident after accounting for the effects of non-inflammatory factors, such as sleep and psychiatric distress. The associations between pain threshold, CRP and sleep problems did not differ based on the presence of fibromyalgia, consistent with other studies advocating a syndrome of widespread pain that spans a spectrum of symptoms and severity, rather than a discrete entity.

These results highlight the multifaceted nature of pain in RA. Physicians and researchers should consider both inflammatory and non-inflammatory factors when evaluating pain in research settings and in the clinic. Future studies are needed to better understand the mechanisms of pain in RA, guide the development of multidisciplinary treatment approaches and test the efficacy of these approaches compared with traditional DMARD therapy.

## Abbreviations

anti-CCP: anti-cyclic citrullinated peptide; BPI-sf: Brief Pain Inventory - short form; CRP: C-reactive protein; DAS28: disease activity score in 28 joints; DMARD: disease-modifying anti-rheumatic drug; ELISA: enzyme-linked immunosorbent assay; HADS: Hospital Anxiety and Depression Scale; ICC: intraclass correlations; MOS: Medical Outcomes Study; NSAID: non-steroidal anti-inflammatory drug; RA: rheumatoid arthritis; RF: rheumatoid factor; SD: standard deviation; TNF: tumor necrosis factor.

## Competing interests

DHS has received salary support through grants from Pfizer Inc., Savient Pharmaceuticals, Millenium Pharmaceuticals, Amgen Inc. and Abbott Laboratories. None of these organizations are financing this manuscript. YCL holds stocks in Merck and Company, Inc. and Novartis. These companies will not gain or lose financially from the publication of this manuscript, either now or in the future. DHS serves as an unpaid member of the executive committee of a Pfizer-sponsored trial on analgesics.

## Authors' contributions

YCL conceived the hypothesis for the manuscript, participated in data collection, conducted the initial statistical analyses, wrote the first draft of the manuscript and had primary responsibility for the manuscript process. LBC, BL and AHF contributed to data management and statistical analyses. ADW, RRE and SMH participated in the design of the study and the interpretation of data. DHS, DJC and EKW participated in study design, analysis and interpretation of data. All authors critically reviewed, contributed and approved the final manuscript.
